# Can Green Algal Plastid Genome Size Be Explained by DNA Repair Mechanisms?

**DOI:** 10.1093/gbe/evaa012

**Published:** 2020-01-23

**Authors:** David Roy Smith

**Affiliations:** Department of Biology, University of Western Ontario, London, ON, Canada

**Keywords:** *Chlamydomonas*, chloroplast genome, genome size, inverted repeat, mutation rate

## Abstract

A major finding in organelle biology over the past decade is that land plant mitochondrial genomes, which are the largest among eukaryotes, can have a “Jekyll and Hyde” mutational pattern: low for synonymous sites, high for intergenic ones. This has led to the theory that double-strand breaks (DSBs) in the intergenic DNA of plant mitogenomes are repaired by inaccurate mechanisms, such as break-induced replication, which can result in large insertions and, thus, could explain why these genomes are so prone to expansion. But how universal is this theory? Can it apply to other giant organelle DNAs, such as the massive plastid DNAs (ptDNAs) of chlamydomonadalean green algae? Indeed, it can. Analysis of the expanded plastomes from two distinct isolates of the unicellular chlamydomonadalean *Chlorosarcinopsis eremi* uncovered exceptionally low rates of synonymous substitution in the coding regions but high substitution rates, including frequent indels, in the noncoding ptDNA, mirroring the trend from land plant mitogenomes. Remarkably, nearly all of the substitutions and indels identified in the noncoding ptDNA of *C. eremi* occur adjacent to or within short inverted palindromic repeats, suggesting that these elements are mutational hotspots. Building upon earlier studies, I propose that these palindromic repeats are predisposed to DSBs and that error-prone repair of these breaks is contributing to genomic expansion. Short palindromic repeats are a common theme among bloated plastomes, including the largest one on record, meaning that these data could have wide-reaching implications for our understanding of ptDNA expansion.

## Introduction

One of my favorite articles from *Genome Biology and Evolution* over the past decade is titled “Plant mitochondrial genome evolution can be explained by DNA repair mechanisms” (Christensen 2013). This paper changed a long-held tenet in the field of organelle genomics—the belief that land plant mitochondrial DNA (mtDNA) has a low mutation rate.

For years, studies consistently recorded low rates of silent-site nucleotide substitution in plant mitochondrial genomes (Wolfe et al. 1987; Palmer and Herbon 1988; Richardson et al. 2013). This, in turn, helped spur the idea that low organelle DNA mutation rates contribute to genomic expansion (Lynch et al. 2006) because plant mitogenomes are typically rich in noncoding DNA. The only problem was that these mitochondrial mutation rate estimates were largely based on alignments of protein-coding sequences (i.e., synonymous substitution rates; *d*_S_) and, therefore, did not necessarily reflect the genome-wide mutational spectrum.

Enter Christensen (2013) who, using entire mitogenome sequences from two ecotypes of *Arabidopsis thaliana*, measured rates of substitution in both coding and noncoding mtDNA. His analyses revealed only a single synonymous substitution in the coding regions, consistent with a low mutation rate. Alignments of the intergenic mtDNA, however, painted a very different picture. In addition to having higher rates of substitution than synonymous sites, they also contained dozens of insertions and deletions (indels) as well as rearrangements. This implied that land plant mtDNA sequence evolution follows a “Jekyll and Hyde” pattern: it is low in coding regions and markedly high in intergenic ones.

How coding and noncoding mtDNA could have such distinctly different rates of silent-site substitution is unknown, but Christensen (2013) argued that it might be linked to different mechanisms of double-strand-break repair, which could be differentially filtered via selection (Christensen 2014; Wu et al. 2019). Coding regions, he proposed, are repaired accurately, likely via homologous recombination or gene conversion, whereas noncoding regions are repaired by inaccurate mechanisms, such as break-induced replication (BIR) or nonhomologous end joining (NHEJ), which can result in rearrangements and large insertions (Christensen 2018). The attraction of this theory is that it explains “two seemingly contradictory features of plant mitochondrial genome evolution—the low mutation rates in genes and the striking expansions of noncoding sequences” (Christensen 2013).

### Parallels between Plant Mitogenomes and Green Algal Plastomes

I took a keen interest in the work of Christensen (2013) because I study the plastid genomes of chlamydomonadalean green algae, which can share certain similarities with plant mitogenomes, including a highly expanded architecture. The Chlamydomonadales boasts dozens of species with plastid DNAs (ptDNAs) in excess of 250 kb (Smith and Lee 2010; Lemieux et al. 2015; Fučíková et al. 2019), including *Volvox carteri* (>525 kb), *Chloromonas rosae* (>710 kb), and *Haematococcus lacustris*, which has the largest plastome on record (1.35 Mb) (Bauman et al. 2018; Smith 2018). Like plant mtDNAs, chlamydomonadalean plastomes are often distended with repeat-rich noncoding DNA and can have unusually low rates of synonymous substitution (Smith and Lee 2010; Del Vasto et al. 2015; Gaouda et al. 2018). But, also like plant mitogenomes, there is a paucity of substitution rate data from the intergenic regions of chlamydomonadalean ptDNAs, largely because the available plastome sequences are too divergent from one another to readily align noncoding sequences (Gaouda et al. 2018). Consequently, it is not yet known if the rate of sequence evolution of bloated chlamydomonadalean ptDNAs follows the same “Jekyll and Hyde” pattern observed in various plant mtDNAs, although some have speculated that it does (Del Vasto et al. 2015).

In the hopes of finding sequences to accurately measure rates of evolution in chlamydomonadalean intergenic ptDNA, I regularly check GenBank for newly uploaded plastid genomes. Recently, I discovered a pair of plastomes that—as described below—provided a novel perspective into plastid mutation rate. These two complete ptDNAs come from distinct isolates of the unicellular, fresh-water chlamydomonadalean *Chlorosarcinopsis eremi*, normally found in desert environments. One comes from *C. eremi* strain UTEX 1186 (GenBank accession MG778185), collected near Phoenix, AZ, in the early 1960s (Chantanachat and Bold 1962), and sequenced as part of a large-scale phylogenetic analysis (Fučíková et al. 2019); the other belongs to strain MKA.28 (GenBank accession MN102114.1), recently isolated from soil in Khabr National Park, Iran (Juy-abad et al. 2018), and sequenced during an organelle genomics project (Juy-abad et al. 2019). The UTEX 1186 and MKA.28 ptDNAs are big (∼298 kb), bloated (∼67% noncoding), AT biased (65%) and, with one small exception (discussed later), have matching gene contents and gene orders (supplementary fig. S1, Supplementary Material online).

When I first download the *C. eremi* plastomes, I was not optimistic that they would be easily aligned; in my experience, chlamydomonadalean strains with the same genus and species names often turn out to be unexpectedly divergent to one another (Smith et al. 2010; Del Vasto et al. 2015). In this case, however, I was pleasantly surprised. A global pairwise alignment of the two genomes using MUSCLE (Edgar 2004) (default settings; implemented through Geneious v10.2.6, Biomatters Ltd.) showed that they are very similar (>98% nucleotide identity). What’s more, all the intergenic regions aligned well. I quickly looked closer at the alignment to see how the coding and noncoding segments differed.

### A “Jekyll and Hyde” Plastome Mutational Pattern

The UTEX 1186 and MKA.28 ptDNAs are extraordinarily similar across their coding regions (table 1). Not a single substitution can be found in rRNAs or tRNAs, and of the 66 protein-coding genes, 57 are identical. Even plastid genes renowned for being highly divergent, such as *ftsH* and *ycf1*, have >99.9% sequence identity. In total, only 15 substitutions exist across 84.5 kb of protein-coding ptDNA, 6 of which are synonymous (*d*_S_ = 0.0005), and none involving 2 or more consecutive sites; a sole coding indel of 21 nt is located within *rpoC2*. Analysis of the sequences outside of genes tells a much different story.

**Table 1:** Nucleotide Divergence between the *Chlorosarcinopsis eremi* UTEX 1186 and MKA.28 Plastomes

	Protein Coding	Noncoding
Alignment length (kb)^a^	84.5	193.2
Nucleotide substitutions	15	445^c^
Synonymous substitutions	6	NA
Substitutions per silent site^b^	∼0.0005	∼0.0025
Indels (accumulative length, nt)	1 (21)	151 (4,114)

Note.—Analyses include only one copy of the large inverted repeat element (supplementary fig. S1, Supplementary Material online). Coding and noncoding substitution rates were calculated using the PAML software suite (Yang 2007) using the same protocols and settings as in Gaouda et al. (2018). Indels: insertions and deletions. NA: not applicable.

a Includes gaps.

b Includes synonymous sites for coding regions and all sites for noncoding regions.

c When counting trinucleotide substitutions events as a single substitutional event, the total number of substitutions is reduced to 333.

Paralleling the work of Christensen (2013), the noncoding ptDNA of UTEX 1186 and MKA.28 abounds with substitutions and indels (table 1). Indeed, 445 nucleotide substitutions are found in 193 kb of aligned intergenic and intronic ptDNA, giving an overall noncoding substitution rate of ∼0.0025, which is five times that of synonymous sites. Unlike for the coding regions, many of the noncoding substitutions occurred consecutively, in runs of three. There are an astounding 56 trinucleotide substitution events, which together encompass 37.8% of all noncoding substitutions. (Note: when counting trinucleotide substitutions as a single substitutional event, the total number of substitutions is reduced to 333.) Equally remarkable, 151 indels are distributed throughout the noncoding ptDNA, ranging from 1 to 1,708 nt (average = 27 nt) (table 1) and accounting for the minor size difference between the UTEX 1186 and MKA.28 plastomes (298,847 nt vs. 298,251 nt). The largest indel, located between *rps9* and *ycf3*, resulted in the complete deletion of *ycf4* from the MKA.28 plastome (supplementary fig. S1, Supplementary Material online); this ∼600-nt gene has been lost from other green algal plastomes (Turmel and Lemieux 2018) but is usually retained in those from the Chlamydomonadales (Fučíková et al. 2019). Altogether, ∼600 mutational events were identified in the noncoding sequences, nearly 50-fold more than those in the coding ones.

**Fig. 1.**
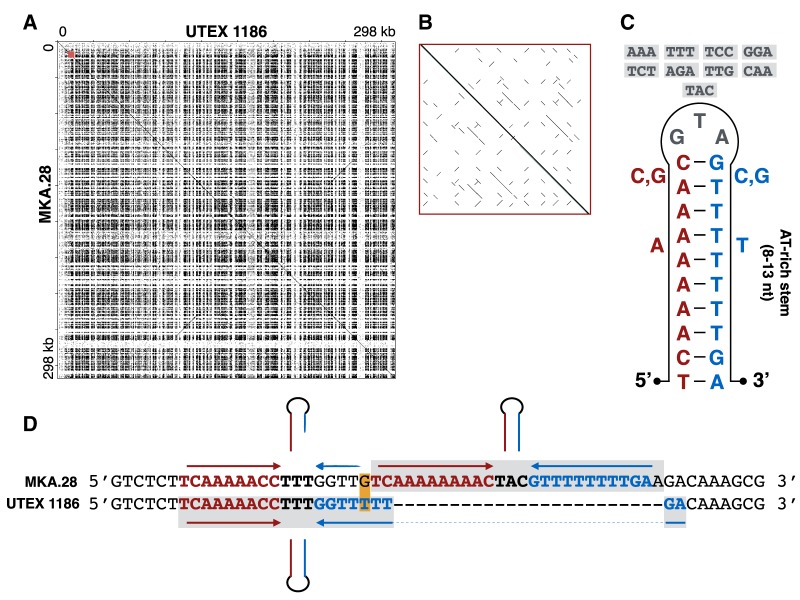
—Palindromic repeats in the *Chlorosarcinopsis eremi* UTEX 1186 and MKA.28 plastomes. (*A*) Dot-plot nucleotide similarity matrix of the UTEX 1186 (*x* axis) and MKA.28 (*y* axis) plastomes; generated with JDotter (Brodie et al. 2004) using a sliding window size of 50. (*B*) Zoomed-in image of dot plot showing better resolution of palindromic elements; corresponds to region highlighted in red on large dot plot. (*C*) Folded hairpin structure of a typical palindromic repeat; nucleotides adjacent to stem represent common additions; boxed nucleotides above loop represent the various sequences involved in palindromic-repeat-associated trinucleotide substitution events. (*D*) Pairwise nucleotide alignment of ptDNA from UTEX 1186 and MKA.28 highlighting an indel event involving a palindromic repeat; palindromes are boxed and their stems (red/blue), loops (black bolded), and hairpin structures are identified; nucleotide substitutions are boxed in orange.

At first glance, these results suggest that there is a certain level of genetic upheaval within the *C. eremi* ptDNA. But upon careful examination, an order emerges from the chaos, and it centers around the repetitive nature of the noncoding regions. The UTEX 1186 and MKA.28 plastomes are populated by hundreds of inverted palindromic repeats (fig. 1*A* and *B*), which can be folded into hairpin/cruciform structures and which share a common motif, consisting of an 8–13-nt AT-rich stem and a 3-nt loop (fig. 1*C*), such as 5′–TCAAAAAAAC**GTA**GTTTTTTTTGA–3′ (palindrome underlined; loop bolded). These palindromes make up ∼50% of the noncoding ptDNA and can even be found in small numbers in some of the coding regions, such as *ftsH*, *rpoC2*, and *ycf1*. Their sequence and location are conserved between UTEX 1186 and MKA.28, but I did find some instances where the copy number and/or orientation of a palindrome differs slightly between the two strains. These small differences yielded big insights into the plastid mutational spectrum.

### Error-Prone Palindromic Repeats

The palindromic repeats in the *C. eremi* plastome are mutational hotspots. Of the ∼150 indels between UTEX 1186 and MKA.28, 53% directly result from the gain or loss of a palindrome (fig. 1*D*), and this does not include indels involving subsections of palindromes. This is true as well for the sole coding indel, reflecting the presence of a 21-nt palindrome within *rpoC2* of MKA.28. Likewise, all but two (96%) of the trinucleotide substitutions are, in fact, microinversion events within palindromes, occurring specifically to the loop portion of the folded hairpin structure; for example, cases where 5′–**GTA**–3′ is found in one strain and 5′–**TAC**–3′ is found in the other (fig. 1*C*). A large proportion (>75%) of the single and dinucleotide noncoding substitutions are also associated with palindromes, occurring within or immediately upstream or downstream (within 10 nt) of palindromic-repeat-mediated indels (fig. 1*D*). Even the 1708-nt deletion from MKA.28, which led to the loss of *ycf4*, is bookended by a pair of palindromes. All told, more than three quarters of the recorded mutational events in the UTEX 1186 and MKA.28 plastomes are tied to palindromes.

Why are the palindromes so prone to mutations? First, it does not appear that the observed differences between UTEX 1186 and MKA.28 are the product of misalignments or misassemblies of repetitive ptDNA. The noncoding regions from these two plastomes contain sufficient nonrepetitive sequence to accurately anchor paired-end Illumina reads to their correct position during genome assembly (Fučíková et al. 2019), and the palindromes themselves generally occur far enough apart—and are distinct enough—that short misalignment errors are unlikely. Nor does it appear that the palindromes are transposable elements. They show no sequence similarity to known transposons and the *C. eremi* ptDNA does encode any proteins typically associated with transposition (but keep in mind that the some of the ptDNA introns encode endonucleases).

There are specific features of the palindromes that might them make catalysts for mutations. For instance, the trinucleotide microinversions are most certainly the consequence of nonhomologous recombination between identical palindromic elements, which could easily occur from small folds in the ptDNA. This phenomenon, which has been documented in the ptDNAs of other species (Kelchner and Wendel 1996), is akin to the commonly seen flipflopping of the single copy regions in plastomes via recombination between the long rRNA-containing inverted repeats (Stein et al. 1986), but on a much smaller scale. It is not as immediately obvious how the palindrome-containing indels are generated but some key themes arise. The gain/loss of palindromes almost always occurs in regions that are themselves part of a palindrome (i.e., a portion of one palindrome jumping into or out of that of another) (fig. 1*D*). Moreover, the specific insertion/deletion sites of these events tend to be immediately adjacent to (or within) the poly-A/T tracts that make up the stem of the folded hairpin, for example, 5′–TCAAAAAAC**AAA**GTTTTTT*GA–3′, where * denotes the insertion/deletion site of a different palindrome.

One explanation for this pattern, which echoes back to the work of Christensen (2013) and others (Wu et al. 2019), is that these palindromic indels arise during the repair of double-strand breaks (DSBs). There is a large and growing body of evidence that short palindromic repeats cause genetic instability and can stimulate DSBs (Bzymek and Lovett 2001; Lobachev et al. 2007; Lu et al. 2015). This can happen, for instance, via replication fork stalling and collapse, resulting in DNA replication-dependent mutagenesis, or through various replication independent, structure-specific means (Lu et al. 2015). Whatever their origin, the repair of DSBs can lead to small or large indels at the break site, as well as other types of mutations (Mehta and Haber 2014). The accuracy of DSB repair, as noted earlier, often reflects the repair pathway that is used, be it error-prone BIR or more accurate homologous recombination (Mehta and Haber 2014; Ramakrishnan et al. 2018).

For *C. eremi*, the idea that palindromic repeats are causing DSBs in ptDNA is supported by the large number of mutations connected to these elements, particularly the indels adjoining or within palindromes—precisely the kinds of regions known to be predisposed to breakage (Mehta and Haber 2014). But why would a DSB beside or within a palindromic repeat results in the partial insertion/deletion of another palindromic element? The answer could be linked to the repair pathway(s) used to restore such a break. Indeed, a DSB at or beside a palindrome, especially when that break is associated with a poly-A/T tract, could lead to short- or microhomology-mediated forms of DSB repair, such as BIR or NHEJ. And given the preponderance of palindromic elements within the *C. eremi* ptDNA, it is easy to envision how a palindrome from an ectopic location could be used as the template for repair, potentially resulting in a short insertion or deletion.

One of the limitations of this work is that I am unable to specifically classify the indel events as insertions or deletions. To do so would require additional ptDNA sequences from other *C. eremi* strains or from a closely related species for which the noncoding regions can be aligned to those of UTEX 1186 and MKA.28. With these kinds of data, it would be possible to see if the palindromic elements are biased toward insertion or deletion mutations, which in turn might also provide further insights into pathways involved in their repair. In this context, it is noteworthy that the number of insertions versus deletions in UTEX 1186 relative to MKA.28 is 99 and 52, respectively. Also, mutation accumulation experiments of *Chlamydomonas reinhardtii* have revealed a bias toward insertions versus deletions in the ptDNA (Ness et al. 2016).

How do these data connect to those on land plant mitogenomes expansion? Christensen presented two hypotheses for the contrasting rates of mtDNA sequence evolution in land plants: 1) different mutation rates owing to different use of DNA repair machinery (Christensen 2013) and 2) different intensities of selection (Christensen 2014). The results described here, at least with respect to green algal ptDNA, point to a third explanation: there is a higher mutation rate in intergenic regions because these regions have tolerated the accumulation of repetitive sequences that are themselves more mutagenic.

### Palindromic Repeats in Other Chlamydomonadalean Plastomes

Palindromic repeats are found in a wide range of organelle genomes (Čechová et al. 2017) and are particularly prevalent in the plastomes of chlamydomonadalean algae (Smith and Lee 2009; Smith et al. 2010; Figueroa-Martinez et al. 2017). In *H. lacustris*, for example, identical palindromic elements have spread throughout the ptDNA and mtDNA, resulting in the extreme expansion of these genomes (Zhang et al. 2019). (Note: the *C. eremi* mtDNA [∼25 kb; GenBank accession MH665695.1] is devoid of palindromic repeats [Juy-abad et al. 2019].) Moreover, the palindromes from other chlamydomonadalean algae often have similar characteristics to those from *C. eremi*, such as an AT-rich stem and a 3-nt loop within the folded hairpin structure (Smith and Lee 2009), although GC-rich hairpins have also been identified in some species (Smith and Lee 2008; Zhang et al. 2019).

If the palindromic ptDNA elements in other chlamydomonadalean species are prone to the same types of mutations found in *C. eremi*, it could help explain why this green algal order has undergone such severe ptDNA expansion, harboring 5 of the 10 largest plastomes sequenced to date. It is telling that in all the explored chlamydomonadalean ptDNAs >250 kb, short palindromic repeats are a near-ubiquitous feature. Likewise, in those that are <250 kb, palindromes are generally absent or present in low quantities. The ptDNAs of *Volvox cateri* and *Volvox africanus* (who shared a common ancestor ∼70 million years ago) exemplify this point. The former is >525 kb and distended with palindromes, whereas the latter is 246 kb and nearly lacks them (Gaouda et al. 2018).

It will be interesting to see if the trends described here for the *C. eremi* ptDNA will be borne out in studies of other chlamydomonadalean algae with large plastomes and in other organelle systems more generally. More detailed data on ptDNA repair pathways in green algae will also be welcomed and could shed additional light on palindromic-repeat-associated errors in *C. eremi*. For now, it is safe to say that the findings of Christensen (2013) are having wide-reaching effects on the field of organelle genomics and that mtDNA and ptDNA mutational patterns are anything but straightforward.

## Supplementary Material

Supplementary data are available at *Genome Biology and Evolution* online.

## Supplementary Material

evaa012_Supplementary_Data
