# Supplementary material for: Can Green Algal Plastid Genome Size Be Explained by DNA Repair Mechanisms?
Source: Genome Biol Evol. 2020 Jan 23;12(2):3797–802. doi: 10.1093/gbe/evaa012 (PMC7043297; doi:10.1093/gbe/evaa012)
Supplement: evaa012_Supplementary_Data [file evaa012_supplementary_data.pdf]

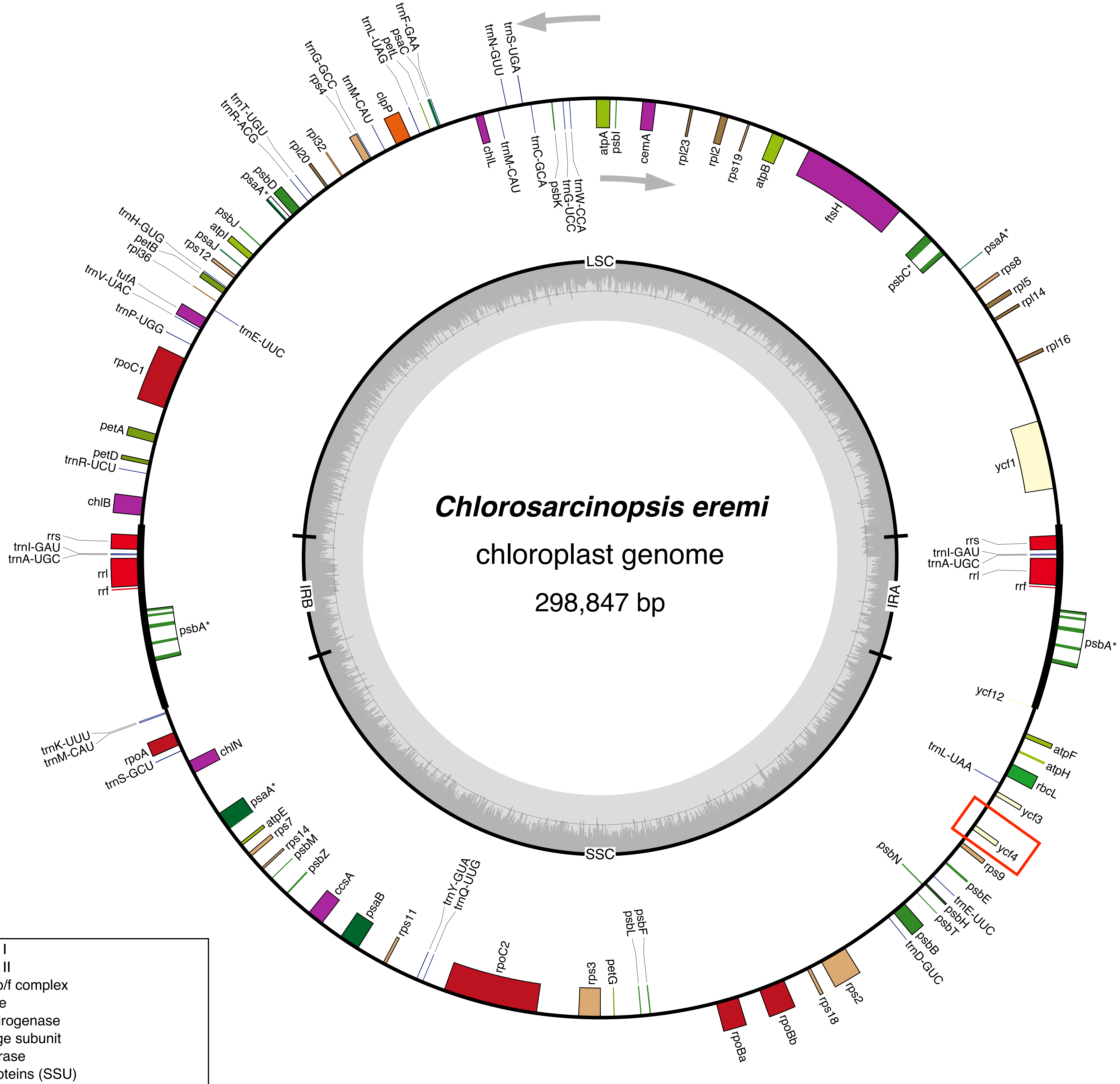

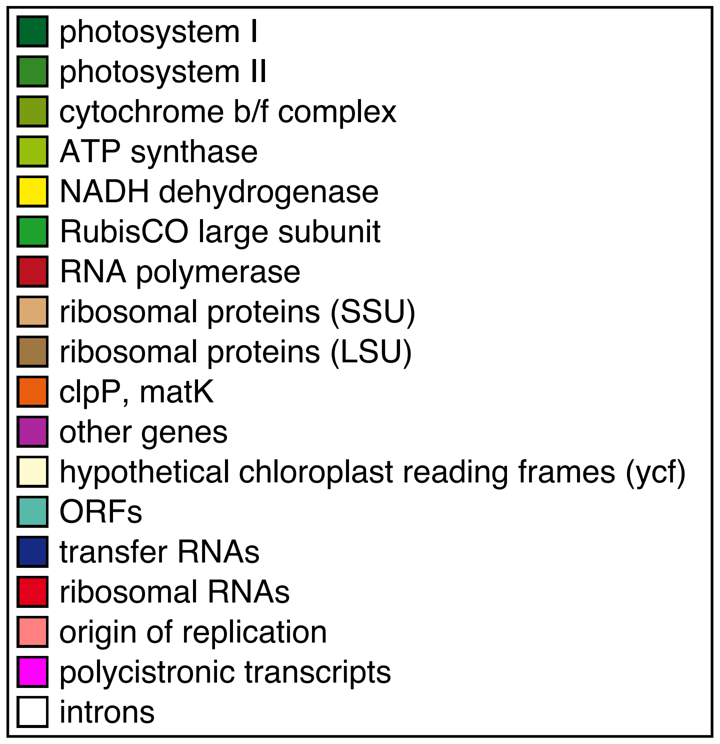

Figure S1. Chloroplast genome map of Chlorosarcinopsis eremi strains UTEX 1186 and MKA.28. Map generated using OrganellarGenomeDRAW (OGDRAW) (Greiner et al. 2019; bioRxiv 545509). Large 1708-nt deletion from MKA.28 is boxed in red.
